# Comparison of single-voxel ^1^H-cardiovascular magnetic resonance spectroscopy techniques for in vivo measurement of myocardial creatine and triglycerides at 3T

**DOI:** 10.1186/s12968-021-00748-x

**Published:** 2021-05-13

**Authors:** Joevin Sourdon, Tangi Roussel, Claire Costes, Patrick Viout, Maxime Guye, Jean-Philippe Ranjeva, Monique Bernard, Frank Kober, Stanislas Rapacchi

**Affiliations:** 1 Aix-Marseille Univ, CNRS, CRMBM UMR 7339, Marseille, France; 2 APHM, Hôpital Universitaire Timone, CEMEREM, Marseille, France

**Keywords:** Cardiac metabolism, CMR spectroscopy, Proton magnetic resonance spectroscopy, 3 T, SLASER, Creatine, Lipids

## Abstract

**Background:**

Single-voxel proton cardiovascular magnetic resonance spectroscopy (^1^H-CMRS) benefits from 3 T to detect metabolic abnormalities with the quantification of intramyocardial fatty acids (FA) and creatine (Cr). Conventional point resolved spectroscopy (PRESS) sequence remains the preferred choice for CMRS, despite its chemical shift displacement error (CSDE) at high field (≥ 3 T). Alternative candidate sequences are the semi-adiabatic Localization by Adiabatic SElective Refocusing (sLASER) recommended for brain and musculoskeletal applications and the localized stimulated echo acquisition mode (STEAM). In this study, we aim to compare these three single-voxel ^1^H-CMRS techniques: PRESS, sLASER and STEAM for reproducible quantification of myocardial FA and Cr at 3 T. Sequences are compared both using breath-hold (BH) and free-breathing (FB) acquisitions.

**Methods:**

CMRS accuracy and theoretical CSDE were verified on a purposely-designed fat–water phantom. FA and Cr CMRS data quality and reliability were evaluated in the interventricular septum of 10 healthy subjects, comparing repeated BH and free-breathing with retrospective gating.

**Results:**

Measured FA/W ratio deviated from expected phantom ratio due to CSDE with all sequences. sLASER supplied the lowest bias (10%, vs -28% and 27% for PRESS and STEAM). In vivo, PRESS provided the highest signal-to-noise ratio (SNR) in FB scans (27.5 for Cr and 103.2 for FA). Nevertheless, a linear regression analysis between the two BH showed a better correlation between myocardial Cr content measured with sLASER compared to PRESS (r = 0.46; p = 0.03 vs. r = 0.35; p = 0.07) and similar slopes of regression lines for FA measurements (r = 0.94; p < 0.001 vs. r = 0.87; p < 0.001). STEAM was unable to perform Cr measurement and was the method with the lowest correlation (r = 0.59; p = 0.07) for FA. No difference was found between measurements done either during BH or FB for Cr, FA and triglycerides using PRESS, sLASER and STEAM.

**Conclusion:**

When quantifying myocardial lipids and creatine with CMR proton spectroscopy at 3 T, PRESS provided higher SNR, while sLASER was more reproducible both with single BH and FB scans.

**Supplementary Information:**

The online version contains supplementary material available at 10.1186/s12968-021-00748-x.

## Introduction

There is an important need to non-invasively quantify cardiac metabolic profiles since myocardial metabolic abnormalities are characteristics of common cardiovascular diseases.

Noninvasive single-voxel proton cardiovascular magnetic resonance spectroscopy (^1^H-CMRS) allows to quantify of intramyocardial lipids and metabolites, such as creatine (Cr). Notably, ^1^H-CMRS has revealed reduced Cr in myocardial infarction [1] and in nonischemic heart diseases [2]. The technique has also shown alteration of myocardial triglyceride (TG) metabolism [3, 4] and TG accumulation in type 2 diabetes [5]. Taken together, reproducible and rapid ^1^H-CMRS will be very useful to follow cardiovascular disease progression and severity [6]: myocardial Cr is associated with the New York Heart Association grade of heart disease [2] and myocardial TG content is related to the cause of disease [7].

Interestingly, ^1^H-CMRS benefits from increased magnetic field strength improving signal-to-noise ratio (SNR) and broadening the spectrum, which eases spectral peak separation. As such, recent ^1^H-CMRS studies have been preferably performed using 3 T CMR scanners [8]. However, most cardiac ^1^H-CMRS at high magnetic field (≥ 3 T) have been limited to using conventional point resolved spectroscopy (PRESS) localization sequences [8, 9]. PRESS is a spin-echo based ^1^H-CMRS sequence allowing relatively short echo times, which is important under the constraints of the beating heart.

PRESS, however, suffers from a magnetic field strength-dependent chemical shift displacement error (CSDE) that shifts the spatial localization of the different measured chemical species. CSDE can be neglected at 1.5 T but becomes problematic at 3 T, such that the international Magnetic Resonance Spectroscopy Consensus Group [10] considered the localization error from PRESS [11] unacceptable for neurological applications. However, in the heart, alternative sequences need yet to be evaluated to provide reliable myocardial spectra at high fields.

Considering cardiac motion beat-to-beat variability, single-voxel ^1^H-CMRS sequences that necessitate 2 acquisitions or more (e.g. ISIS [12], SPECIAL [13]) have not been considered in the following. Thus, the following single-shot single-voxel ^1^H-CMRS sequences were considered eligible for reliable cardiac ^1^H-CMRS: PRESS, semi-adiabatic Localization by Adiabatic SElective Refocusing (sLASER) and STimulated-Echo Acquisition Mode (STEAM).

The Magnetic Resonance Spectroscopy Consensus Group recommended the use of sLASER at high fields (≥ 3 T) for brain [14] and musculoskeletal [15] applications to improve reproducibility of data acquisition and metabolite quantification. sLASER has the advantages to provide a considerably lower CSDE with a sharper voxel localization compared to PRESS [16,17]. sLASER also boasts a low sensitivity to B0 and B1 inhomogeneities thanks to adiabatic radiofrequency (RF) pulses and reduced J-coupling from quadruple refocalization. However, it suffers from longer minimum echo time (TE) and higher RF energy deposit being directly limited by specific absorption rate (SAR) restrictions at high fields. STEAM has the advantage to allow for short TE. Also, the 90° RF pulses used in STEAM can be performed with a larger bandwidth, covering the spectrum more homogeneously with lower energy deposit. Nevertheless, the simulated echo signal amplitude is two times lower than that of spin echoes, and STEAM is penalized by higher sensitivity to B0 and B1 inhomogeneity.

In this study, we aim to compare the three single-voxel ^1^H-CMRS techniques PRESS, sLASER and STEAM in the context of CMRS at 3 T. Both scenarios of breath-hold (BH) CMRS and free-breathing (FB) CMRS acquisitions were considered. The set target has been to reliably quantify intramyocardial lipid storage and Cr content.

## Methods

### Voxel localization precision

Theoretical CSDE for each sequence has been computed for each CMRS sequence using the following equation:

$$CSDE=\frac{\Delta f}{BW}$$

where Δ*f* is the frequency difference of two resonances (Hz) of interest and BW is the bandwidth (Hz) of the slice-selective refocusing RF pulse. Considering the voltage calibration for a typical torso exam at 3 T while minimizing TE, the duration of refocusing RF pulses were set to 7.08 ms for PRESS and 12 ms for sLASER (adiabatic full passage first-order hyperbolic secant pulses with a time-BW product of 20) to guarantee maximal longitudinal magnetization inversion. The 90° pulses for STEAM were set to 2.56 ms using the default asymmetrical RF shapes [18]. Corresponding spectral bandwidths were BW = 1150, 1700 and 2200 Hz for PRESS, sLASER and STEAM respectively.

### CMR protocol prior to CMRS acquisitions

Measurements were performed on a 3 T CMR scanner (Verio, VB17 software, Siemens Healthineers, Erlangen, Germany) using a dedicated cardiac 32-channel receive array (InVivo Corporation, Gainesville, Florida, USA). Prior to CMRS acquisitions, protocols included electrocardiogram (ECG)-triggered spatial localizer images in at least 2 perpendicular planes cutting through the center of the voxel of interest, followed by ECG-triggered automatic projection-based first-order B_0_ shimming in the same voxel with FAST(EST)MAP [19]. Iterative shimming was performed until stability of the results (2 or 3 repetitions) was achieved.

### CMRS protocol

The CMRS acquisition voxel size was 9 mL, placed in vivo in the interventricular septum, with typical dimensions of 15 × 20 × 30 mm^3^ unless adjusted for a thinner septum. Single-voxel CMRS was performed using PRESS, sLASER [20, 21] and STEAM [18] provided by Center for Magnetic Resonance Reseach (CMRR, VB17, available at https://www.cmrr.umn.edu/spectro/). All three sequences included an outer volume suppression (OVS) preparation with six 80 mm-thick saturation bands placed with a 5 mm gap from the voxel edges. We used the minimum TE feasible with each sequence (23.96, 5.4, 64.24 ms for PRESS, STEAM and sLASER, respectively). STEAM mixing time (TM) was 26.5 ms. Due to SAR restrictions, the minimum repetition time (TR) for water-saturated PRESS and sLASER was 1.1 s. ECG triggering led to a repetition every 2-RR for most exams whence RR duration was shorter than 1.1 s, thus TR was equal to 2-RR. Suppression of water (W) signal was realized using variable pulse power and optimized relaxation delays (VAPOR) [18].

### CSDE evaluation using a phantom experiment

**Fig. 1:**
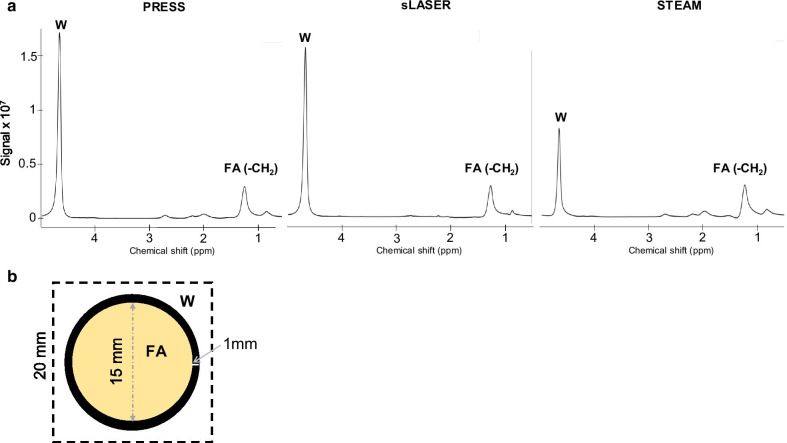
**a** Spectra from the homemade phantom containing fat acquired with three different sequences (PRESS, sLASER and STEAM) showing the resonances of water (W, 4.7 ppm) and fatty acid (FA, 1.2 ppm). **b** Acquisitions were realized without water suppression using a voxel of 20 × 20 × 30mm^3^ (dashed line) that surrounded the fat-containing cylindrical tube. Excitation was prescribed through-plane, and both refocusing pulses were prescribed in-plane, according to the dotted box

CMRS data were acquired on a homemade phantom containing fat (vegetable oil) and water compartments for in vitro comparison of sequences. A 30 × 20 × 20 mm^3^ voxel was placed on a fat-containing cylindrical tube with r = 7.5 mm radius that was surrounded by water (Fig. 1b). The three ^1^H-CMRS sequences described above were used without water suppression. Based on voxel geometry prescription, the fat/water ratio was expected to be:

$$\frac{FA}{W}=100\left(\frac{{7.5}^{2}\uppi }{{20}^{2}-\left({8.5}^{2}\pi \right)}\right)=102\%$$.

### In vivo experiments

Written informed consent for the study inclusion was provided by all participants. The study was approved by the local institutional ethics review board. A total of 10 healthy subjects (8 men; 35.9 ± 11.4 years; range: 25–60 years; body mass index (BMI): 23.6 ± 2.7 kg/m^2^ and heart rate: 69 ± 13 beats per minute) were examined in supine position. The 15 × 25 × 30 mm^3^ voxel was placed in the interventricular septum in diastole on the cine 4 chamber and mid-ventricular short-axis views. All CMRS measurements were assessed in the exact same volume of interest (VOI) using the three sequences described above with or without water suppression. First, for each sequence, 8 water-suppressed averages were acquired during a single BH of typically 12–16 s. Second, single non-water-suppressed spectra were acquired in separate short BHs. Third, 100 repetitions water-suppressed spectra were acquired during FB. Fourth, the BH 8 averages were repeated for reproducibility. PRESS and sLASER were randomly played first to confirm proper voxel positioning. Following sequences order was completely randomized. ECG triggering was determined from the manufacturer’s vector ECG signal, with the signal sampling being placed at end-diastole.

### CMRS data processing

A custom Python-based software was used to post-process raw CMRS data [22]. The signal processing included: automatic channel-by-channel signal 0th and 1st order phasing, Single Value Decomposition (SVD) channel recombination, zero-filling, individual spectra frequency realignment and a 10 Hz-damping apodization.

### Free-breathing and retrospective gating

Individual CMR spectra were systematically and automatically analyzed by estimating amplitude, linewidth, chemical shift, and phase variations of the residual water peak across the acquired series. In the case of FB-acquired CMRS data, a two-stage retrospective gating approach was employed. The first stage consisted in discarding individual spectra based on their peak linewidth. The maximum peak linewidth threshold was automatically defined by a home-made algorithm that evaluated the fatty acid (FA) SNR using step-wise increases of the threshold that continued to increment only if SNR was maintained or increased while reducing the resulting peak linewidth. The second stage consisted in rejecting any individual spectra for which the estimated phase shift deviated more than 0.6 × standard deviation (SD) estimated over the whole scan, as previously described [23]. The number of individual scans discarded because of excessive peak linewidth or phase variations were extracted for later use.

### SNR and linewidth estimations

The linewidth (LW) of the water resonance was measured as the FWHM (Full Width at Half-Max) of the peak at 4.7 ppm in the non-water-suppressed spectra. Phantom FA content as well as myocardial Cr, FA and TG were analyzed from water-suppressed spectra. SNR was calculated as the ratio of the Cr peak at 3 ppm or FA at 1.2 ppm divided by the SD of noise measured between − 3 and − 1 ppm. Data with LW of water > 35 Hz and peaks with a SNR < 5 were excluded from the final analysis.

### Quantification

Processed data were quantified using home-made Python software described elsewhere [22]. The fitting algorithm strongly relies on a time-domain CMRS model consisting of a linear combination of numerically computed metabolite spectra. The pyGAMMA simulation library [24] was employed to simulate tCr (3.027 ppm), myocardial TG and TMA (3.183 ppm) using a spin-echo acquisition. Myocardial TG was modeled using 6 Gaussian components in total [8] and consisted of two groups: FA at 0.9, 1.3 and 1.6 ppm and unsaturated FA (UFA) at 2.1, 2.3 and 2.8 ppm. The CMRS model was numerically adjusted to the data using a non-linear least-squares optimization algorithm resulting in a relative concentration and frequency shift estimates for each metabolite as well as an overall linewidth damping and phase shift. Cramér-Rao Lower Bounds (CRLB) were also estimated considering a noise level measured on the unprocessed unfiltered raw data.

The ratios FA/W and Cr/W were given as percentages. For in vitro experiments, T1 relaxation times were much shorter than TR such that T1 saturation correction was not required. T2 was estimated in our phantom by obtaining spin-echo spectra at TE = 23.96, 28.96, 33.96, 38.96, 43.96, 48.96, 53,96, 58,96, 63,96 and 68.96 ms. T2 was 57.37 ms for FA at 1.3 ppm and 249.5 ms for water, respectively.

For in vivo experiments, ratios were given after T1 and T2 correction with T1 found in literature as 0.35 s for lipids [25,  26], 1.20 s for water [26] and estimated as 1.00 s for Cr from studies in skeletal muscle [25, 27]. Values of T2 were taken from the literature as 89 ms for lipids [25, 26] and estimated as 135 ms for Cr from studies conducted on skeletal muscle [25, 27]. T2 relaxation of water was measured in the myocardium of one subject as 44 ms from multi-TE PRESS measurements, which matched previously published values [28].

The following equation was used for T1 and T2 correction:

$${\text{S}}^{*}_{{\text{N}}} = {\text{ S}}_{{\text{N}}} \times \, \left[ {{1}/\left( {{1} - {\text{exp}}\left( { - {\text{TR}}/{\text{T1}}_{{\text{N}}} } \right)} \right)} \right] \, \times {\text{ exp}}\left( {{\text{TE}}/{\text{T2}}_{{\text{N}}} } \right),$$

where S_N_ is the relative concentration estimate in the VOI for either W, Cr, FA or UFA, S_N_^*^ is the corrected S_N_ for the given biomolecule, TR is the pulse sequence repetition period, T1_N_ and T2_N_ are the spin lattice relaxation and the spin–spin relaxation of the biomolecule, and TE is the echo time. Signals from STEAM, S_N_^**^, were also corrected for the mixing time TM using the following equation:

$${\text{S}}^{**}_{{{\text{N }} = }} {\text{S}}^{*}_{{\text{N}}} \times {\text{ exp}}\left( {{\text{TM}}/{\text{T1}}_{{\text{N}}} } \right).$$

We used the W signal from the water-unsuppressed spectra as a concentration of reference to quantify myocardial Cr, FA and TG:

- Concentration of Cr was calculated as already published [2, 29, 30] and according to the following equation:
  $$\left[ {{\text{Cr}}} \right] \, = { 2}/{3 } \times \, \left[ {\text{W}} \right] \, \times \, \left( {{\text{S}}^{*}_{{{\text{Cr}}}} /{\text{ S}}^{*}_{{\text{W}}} } \right)$$
  The ratio 2/3 accounts for the number of protons on water and the N-methyl resonance group of creatine.
- Myocardial FA was estimated from the peak at 1.3 ppm [7]. Concentration of TG was calculated according to the following equation:
  $$\left[ {{\text{TG}}} \right] \, = { 2}/{28 } \times \, \left[ {\text{W}} \right] \, \times \, \left( {{\text{S}}^{*}_{{{\text{TG}}}} /{\text{ S}}^{*}_{{\text{W}}} } \right)$$
  The ratio 2/28 accounts for the number of protons on water and the estimated average of N-methyl resonance group in FA.
- Myocardial TG was quantified as the sum of the amplitudes of its components that include peaks at (0.9, 1.3, 1.6 ppm) and UFA (2.1, 2.3 and 2.8 ppm) [8, 31]. Concentration of TG was calculated according the following equation:
  $$\left[ {{\text{TG}}} \right] \, = { 2}/{93 } \times \, \left[ {\text{W}} \right] \, \times \, \left( {{\text{S}}^{*}_{{{\text{TG}}}} /{\text{ S}}^{*}_{{\text{W}}} } \right)$$

The ratio 2/93 accounts for the number of protons in water and the average sum of proton resonance group of TG (Methyl, methylene, β-carboxyl, α-olefinic, α-carboxyl and diacyl) [32].

Myocardial tissue water content (55.5 mol/L) was taken as 72.7% by weight [33]:

$$\left[ {\text{W}} \right]{\text{ expressed in }}\upmu {\text{mol}}/{\text{g }} = { 55}.{5 } \times \, 0.{727 } \times { 1}000$$

### Statistics

Data are expressed as mean ± SD. Linear regression analysis and Pearson correlation coefficient were used to determine the repeatability between two BH measurements and to determine the relationship between measurements assessed during one BH and over FB. Bland–Altman tests were also performed to check agreement between the two measurements (BH1 vs. BH2). Test–retest intraclass correlation coefficient (ICC), an index of concordance for dimensional measurements [34], was used to compare the repeatability between two BH measurements. The ICC ranges between -1 (no reliability) and 1 (maximum reliability) with values less than 0.5, between 0.5 and 0.75, between 0.75 and 0.90 and higher than 0.90 considered as poor, moderate, good and excellent reliability [34]. Multiple comparison was performed by one-way analysis of variance followed by a Student t-test to check differences between acquisition methods. A *p* value < 0.05 was considered statistically significant.

## Results

### Voxel localization at 3 T and in vitro phantom experiment

The three main ^1^H-CMRS sequences used to measure fatty acid content in the phantom were PRESS, sLASER and STEAM (**Fig. ****1**) with the refocusing RF pulses’ BW set to 1150, 1700 and 2200 Hz, respectively. Therefore, theoretical in-plane CSDE was 36, 25 and 19% for PRESS, sLASER and STEAM, respectively.

In our phantom experiments, STEAM yielded the lowest SNR: SNR (FA) was 600 vs. 998 and 1195 for STEAM, PRESS and sLASER, respectively and matching SNR (W) were 1688 vs. 5863 and 6317. Table 1 summarizes the experimental ratios obtained with PRESS, sLASER and STEAM after T1 and T2 correction. sLASER supplied a FA/W ratio on par with the theoretical ratio (112% vs. 102%). PRESS had the lowest FA/W ratio with a 28% difference, as expected, and STEAM overestimated the FA content with an increase of 27% as compared to the theoretical ratio despite a lower CSDE.

**Table 1:** Characteristics of ^1^H-MRS sequences PRESS, sLASER and STEAM and results obtained on phantoms

	PRESS	sLASER	STEAM
BW (Hz)	1150	1700	2200
CSDE (%)	36	25	19
FA/W exp (%)	74	112	129
[FA/W]th—[FA/W]exp	− 28	10	27

FA/W (%) exp represents the ratio calculated experimentally on the fat-containing cylindrical tube, [FA/W]th represents the theoretical ratio based on volumes. BW: Bandwidth, CSDE: Chemical shift displacement error in one direction

### In vivo spectral quality

**Fig. 2:**
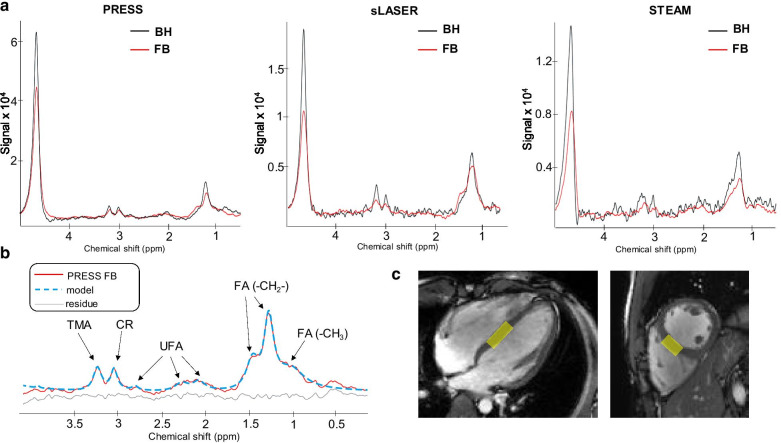
**a** Examples of spectra obtained on the same healthy subject with the same volume of interest placed on the interventricular septum (**c**). Black curves represent the spectra assessed during one breath hold (BH). Red curves represent the spectra assessed during free breathing (FB). BH spectra tended to exhibit higher noise levels than FB. **b** The fitting processing was designed to quantify trimethyl amide (TMA, 3.2 ppm), creatine (CR, 3.1 ppm) and the total myocardial triglyceride (TG) resonance, which includes fatty acids (FA, 0.9, 1.3, and 1.6 ppm) and unsaturated fatty acids (UFA, 2.1 and 2.3 and 2.8 ppm). Residual signal enables to evaluate the adequacy of the model

Figure 2 shows examples of in vivo spectra from the three evaluated sequences. The volume of interest was defined from perpendicular cine images in diastole (Fig. 2c). Septum thinning led to elongated voxel dimensions. The black curves represent spectra assessed during one BH (8 averages). The overlaid red curves represent spectra assessed during free breathing (100 averages) after the postprocessing rejection of 52 ± 9%, 46 ± 8% and 44 ± 10% of averages for PRESS, sLASER and STEAM, respectively. No significant difference was found between the percentages of data rejection. Individual scans were discarded because of excessive phase distortions in 90% and 50% of the subjects for PRESS and semi-LASER, respectively. Other CMRS data quality parameters such as peak amplitude, linewidth and chemical shift exceeded thresholds respectively in 20% and 60% of the subjects for PRESS and semi-LASER. The fitting processing (Fig. 2b) was designed to quantify trimethyl amide (TMA, 3.2 ppm), creatine (CR, 3.1 ppm) and the total myocardial TG resonances, which include fatty acids (FA, 0.9, 1.3, and 1.6 ppm) and unsaturated fatty acids (UFA, 2.1 and 2.3 and 2.8 ppm). Peaks at 2.1, 2.3 and 2.8 ppm were generally less spectrally resolved in vivo with sLASER.

**Fig. 3:**
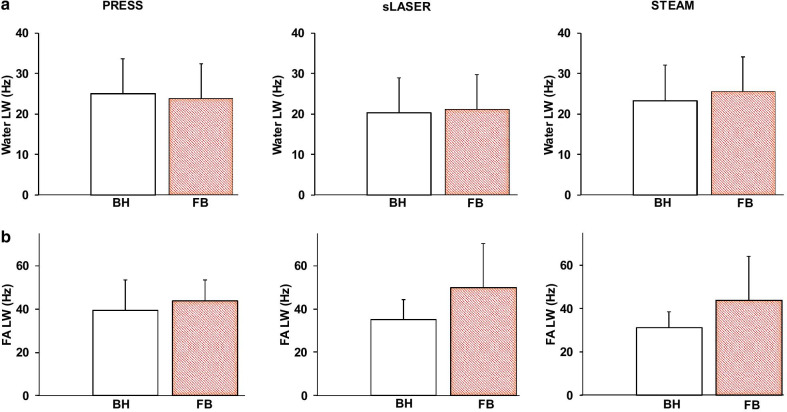
**a** Average of water linewidths measured from 10 subjects from non-water suppressed spectrum assessed during BH (white) and FB (red) for PRESS, sLASER and STEAM. **b** Average of FA linewidths measured from 10 subjects from water suppressed spectrum assessed during BH (white) and FB (red) for PRESS, sLASER and STEAM. Data expressed as mean ± SD. PRESS: BH, n = 20 & FB n = 10; sLASER: BH, n = 20 & FB n = 10; STEAM: BH, n = 9 for Cr, n = 14 for FA & FB n = 8 for both Cr and FA

**Fig. 4:**
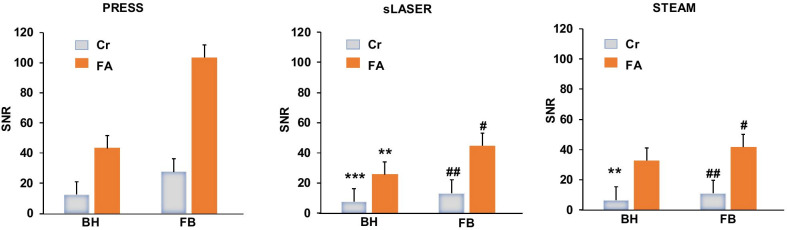
Graphs of mean Cr (blue) and FA (orange) SNR for PRESS, sLASER and STEAM. Data expressed as mean ± SD. PRESS: BH, n = 20 & FB n = 10; sLASER: BH, n = 20 & FB n = 10; STEAM: BH, n = 9 for Cr, n = 14 for FA & FB n = 8 for both Cr and FA. *: *p* < 0.05; **: *p* < 0.01 and ***: *p* < 0.001 compared with the same metabolite (Cr or FA) measured in BH with PRESS. #: *p* < 0.05 and ##: *p* < 0.01 compared with the same SNR metabolite (Cr or FA) measured during FB with PRESS. No difference was found between sLASER and STEAM

The general spectral quality highly depended on the estimated LW of the unsuppressed water signal which ranged around 20 Hz (Fig. 3a) while the lipid LW ranged around 40 Hz (Fig. 3b) using either the BH or FB methods. Despite a slightly larger water LW using PRESS, no significant difference was found between water LW measured with PRESS, sLASER and STEAM. Surprisingly, FB yielded a slightly larger lipid LW for sLASER and STEAM with no statistical difference. The PRESS sequence gave significantly higher SNR (Fig. 4) of metabolite signals compared to sLASER and STEAM for measurements done under BH (SNR Cr: 12.4 ± 5.7 vs. 7.7 ± 2.4, *p* < 0.001 vs. 6.4 ± 2.9 *p* < 0.01 and SNR FA: 43.2 ± 23.8 vs. 25.7 ± 17.0, *p* < 0.05 vs. 32.6 ± 22.3, n.s for PRESS, sLASER and STEAM) and for those acquired during FB (SNR Cr: 27.5 ± 15.0 vs. 13.3 ± 5.4, *p* < 0.01 vs. 11.0 ± 4.2, *p* < 0.01 and SNR FA: 103.2 ± 75.0 vs. 44.5 ± 23.2, *p* < 0.05 vs. 41.5 ± 33.5, *p* < 0.05 for PRESS, sLASER and STEAM). When accounting for T2 signal decay, the T2-corrected SNR between PRESS and sLASER were on par for BH acquisitions (56.5 vs 52.3 respectively), but still differed for FB acquisitions (135.0 vs 91.6 for PRESS and sLASER respectively). Nevertheless, measurements performed during FB enabled a higher SNR for Cr and FA as compared with those assessed during BH except for the measurement of FA realized with STEAM.

As already observed in Fig. 2a, STEAM was not sufficiently robust in our study to reliably measure Cr. Over the 10 exams, STEAM acquired data presented acceptable SNR (above 5) and LW (below 35 Hz) evaluations for only 6 measurements of Cr during the first BH acquisition, 3 during the second BH acquisition and 7 during FB. Similarly, 8 measurements of FA during the first BH, 6 during the second BH and 8 for the FB with STEAM were analyzable.

### Repeatability

**Fig. 5:**
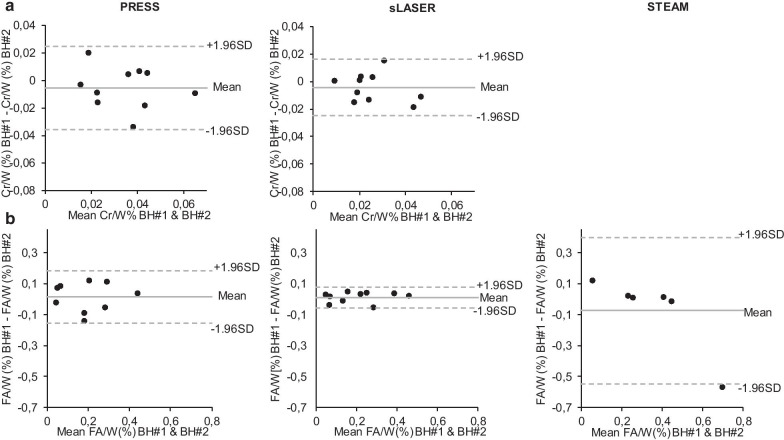
Bland–Altman plots showing reproducibility between two repeated ^1^H-CMRS measures within a single CMR session in the 10 subjects regarding myocardial creatine (**a**) and fatty acids (**b**). Metabolite contents were acquired using two single breath-hold methods with PRESS (n = 10), sLASER (n = 10) and STEAM (n = 6). The solid line represents the mean of differences between levels obtained with the repeated BH methods, the dashed lines indicate the confidence intervals ± 1.96 SD. Peak at 1.2 ppm was used to calculate FA levels. BH: breath hold; Cr: creatine; FA: fatty acids; SD: standard deviation; W: water

The Bland–Altman plots (Fig. 5) demonstrate that the differences for Cr and FA layed within the mean ± 1.96 SD in the healthy subjects with PRESS and sLASER, suggesting that our results had an acceptable reproducibility [35]. Moreover, we observed a smaller confidence interval with sLASER suggesting less variability and better reproducibility. STEAM provided less reproducibility with one measurement outside the limit of agreement and larger confidence intervals (Fig. 5b). Bland–Altman analysis for the evaluation of the agreement between myocardial Cr levels is not shown for STEAM since only three points could be analyzed.

**Fig. 6:**
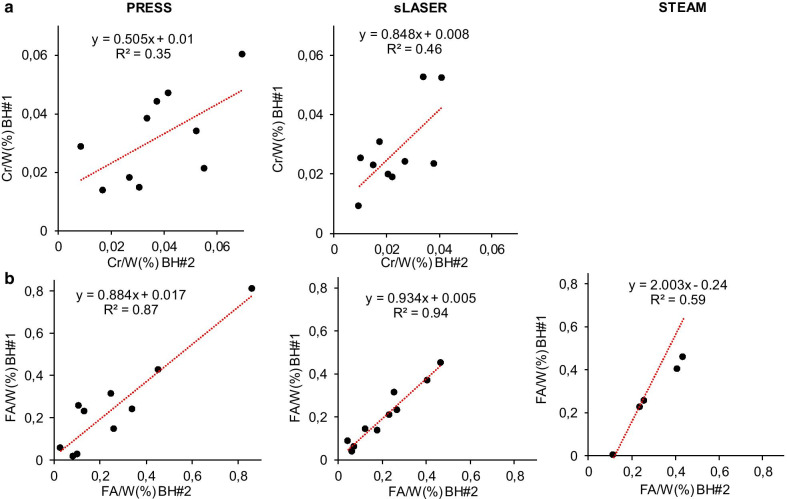
Linear regression analysis showing the correlation between myocardial creatine (**a**) and fatty acids (**b**) content relative to water in two acquisitions in breath-hold with PRESS (n = 10), sLASER (n = 10) and STEAM (n = 6). BH: breath-hold; Cr: creatine; FA: fatty acids; FB: free-breathing; W: water

Linear regression analysis between the two BH showed a better correlation between myocardial Cr content measured with sLASER compared to PRESS (*r* = 0.46; *p* = 0.03 vs. *r* = 0.35; *p* = 0.07) (Fig. 6a). Accordingly, sLASER provided better reliability between measurements as compared to PRESS as shown by the test–retest ICC (0.65 vs. 0.58, both considerated as moderate). PRESS and sLASER had similar slopes of regression lines and similar *r* (*r* = 0.87; *p* < 0.001 vs. *r* = 0.94; *p* < 0.001) with regard to FA and an excellent reliability between these measurements (ICC: 0.94 vs. 0.97) (Fig. 6b). STEAM was the method with the lowest correlation (*r* = 0.59; *p* = 0.07) and moderate reliability between measurements (ICC = 0.52).

### Correlation between BH and FB

**Fig. 7:**
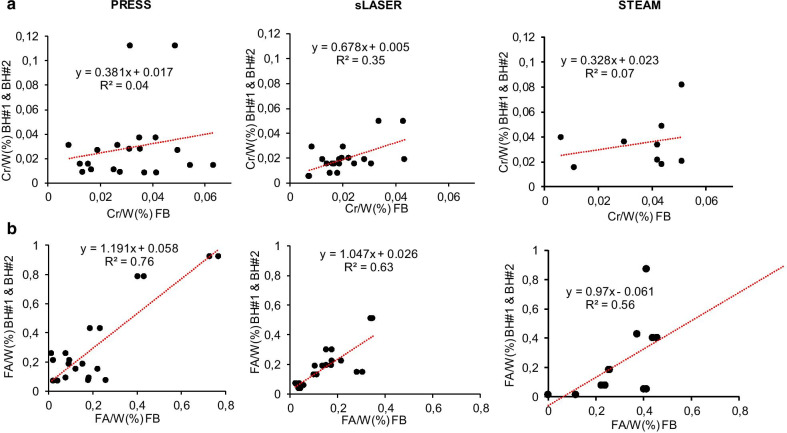
Linear regression analysis showing the correlation between myocardial creatine (**a**) and fatty acid (**b**) content relative to water in acquisitions realized in breath-hold and free-breathing with PRESS and sLASER. BH: breath-hold; Cr: creatine; FA: fatty acids; FB: free-breathing; W: water

Strong correlations were confirmed between myocardial FA levels obtained in a single BH and FB with PRESS (*r* = 0.76, *p* < 0.001), sLASER (*r* = 0.63, *p* < 0.001) and STEAM (*r* = 0.56, *p* < 0.01) (Fig. 7b). A solid correlation was confirmed between myocardial Cr levels obtained in a single BH and FB with sLASER (*r* = 0.35, *p* < 0.01) (Fig. 7a). However, there was no correlation between myocardial Cr obtained in single BH or FB with PRESS (r = 0.04, n.s) and with STEAM (*r* = 0.07, n.s).

### Concentrations of Cr and TG

**Fig. 8:**
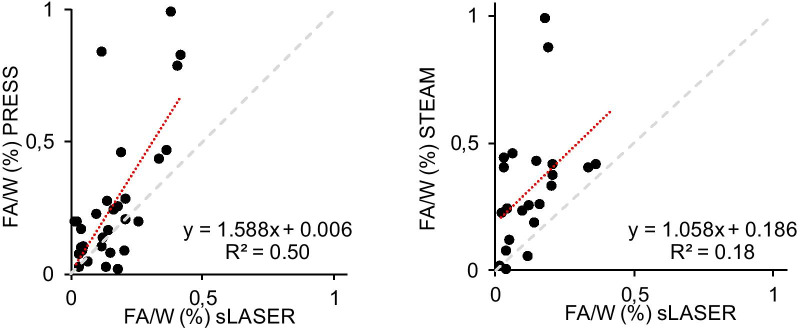
Ratio of signals from fatty acids to water given by PRESS and STEAM compared to that given by sLASER. The dashed red line represents the linear regression analysis between ratio measured with PRESS and sLASER or STEAM and sLASER. The dashed grey lines indicate unity

The mean myocardial Cr, FA and TG content measured relative to water in healthy subjects as well as [Cr], [FA] and [TG] are summarized in Table 2. Cr level content and [Cr] were significantly lower when measured during BH with sLASER as compared to PRESS and STEAM (*p* < 0.001 and *p* < 0.05, respectively). In addition, the measurement of [FA] with PRESS and STEAM (*p* < 0.05) was higher as shown in Fig. 8. There was no difference between the measurements of TG content with the three methods.

**Table 2.** ^1^H-CMRS data and concentrations of metabolites of the study participant. Data expressed as mean ± SD

	PRESS		sLASER		STEAM	
	BH 1 & 2	FB	BH 1 & 2	FB	BH 1 & 2	FB
Cr/W (%)	0.032 ± 0.015**	0.029 ± 0.031	0.021 ± 0.010	0.020 ± 0.012	0.035 ± 0.020*	0.035 ± 0.016
[Cr] µmol/g	9.33 ± 4.51**	8.44 ± 8.88	6.94 ± 3.39	5.29 ± 3.32	9.41 ± 5.28*	13.02 ± 17.62
FA/W (%)	0.22 ± 0.22	0.31 ± 0.30	0.15 ± 0.10	0.18 ± 0.14	0.35 ± 0.23*	0.29 ± 0.31
[FA] µmol/g	7.41 ± 6.80	10.50 ± 9.29	5.96 ± 3.96	5.27 ± 4.04	10.08 ± 6.47*	8.13 ± 8.17
TG/W (%)	0.68 ± 0.50	0.97 ± 0.82	0.48 ± 0.33	0.45 ± 0.43	0.91 ± 1.06	0.67 ± 0.44
[TG] µmol/g	5.87 ± 4.33	8.46 ± 7.16	4.12 ± 2.88	3.90 ± 3.74	7.55 ± 8.50	5.86 ± 3.81

^*^p < 0.05 vs. sLASER BH 1& 2; ** *p* < 0.01 vs. sLASER BH 1 & 2. PRESS, n = 20 (BH) and n = 10 (FB); sLASER, n = 20 (BH) and n = 10 (FB); STEAM, n = 9 (BH) and n = 8 (FB) for Cr; n = 14 (BH) and n = 8 (FB) for FA + TG

## Discussion

This study compares three different sequences for the specific application of cardiac ^1^H-CMRS at 3 T, using both BH and FB acquisitions. In this context, this study demonstrates the feasibility of using the ^1^H-CMRS sequence sLASER for Cr and TG measurement in cardiology. To the best of our knowledge, this is the first study reporting the use of sLASER for CMRS. We show here that the utilization of sLASER allows reproducible metabolite quantification when compared to other CMRS sequence candidates.

First, chemical shift displacement was considered as a major bias. In the specific case of measuring only one or two peaks, chemical shift displacement could be addressed by displaying the corresponding shifted VOI at the console. However, considering the tedious task to manually place the VOI at the same position twice or thrice in a clinical workflow, and the persistent limitation when studying the entire spectrum, we believe CSDE to be a drawback for PRESS to be acknowledged at high field. The experiment on a fat phantom was designed to verify water-fat contamination. As predicted from sequence designs, PRESS supplied the lowest FA/W ratio, due to a displacement of the VOI between fat and water sampling; sLASER demonstrated minimal contamination owing to the use of adiabatic inversion pulses with larger BW; on the contrary, FA/W ratio measured with STEAM was over-estimated despite a low CSDE. This bias might originate from the use of asymmetrical RF pulses, which minimizes RF duration at the cost of reduced transition sharpness (i.e. degraded voxel definition) and oscillating signal within the passband (i.e. intravoxel signal inhomogeneity). Hence, sLASER reduces CSDE and allows a more accurate measurement that leads to myocardial spectra with less contamination from ventricular blood and epicardial adipose tissue. These results align with a report showing that CSDE in the form of extracranial lipid signal were considerably reduced in sLASER compared to PRESS [17] at high field. Further developments could focus on the implementation of shorter adiabatic inversions to reduce sLASER TE, which remains particularly long (64 ms) for cardiac applications and accounted for most of the SNR loss when compared to PRESS in BH acquisitions. The additional under-performances of sLASER reported in FB acquisitions could be manifold: possibly sLASER sensitivity to motion due to its elongated TE [36] and/or an accrued impact of breathing motion and B0 variations on sLASER measurements. As an alternative, the STEAM sequence could be purposely designed to obtain more reliable results with more stable in vivo spectra. Nevertheless, PRESS flaws are bound to remain as the sequence was already well optimized for the heart in this study.

Second, BH CMRS reproducibility was evaluated for the measurement of myocardial Cr and TG. Due to the limitation of minimum TR from SAR restrictions being higher than most R-R durations, BH acquisitions were set to acquire 8 averages to maintain the apnea time below 15 s. RF energy deployment was necessarily high due to the concurrent use of VAPOR water suppression with high-voltage and short RF pulses. A cardiac-dedicated optimization of water suppression that allows the minimum TR to fit within one heartbeat could certainly double the number of averages acquired in a single BH. Breath-holding is often used for respiratory motion compensation in routine CMR and could be easily repeated and reproduced without any specific set-up [37]. Eventually, excellent test–retest for TG measurements was found (ICC > 0.9 for PRESS and sLASER). These results establish the reliability and clinical readiness of CMR spectroscopy. Previously, Rial et al*.* also observed an excellent agreement of the lipid levels assessed in a single BH (4 acquisitions) and in multiple‐BHs (35 acquisitions) by using STEAM at 3 T [26].

Third, FB CMRS was compared to BH acquisitions for the measurement of myocardial Cr and TG. Our study found similar values between FB and BH for Cr/W (using sLASER) and for TG/W ratios (using either PRESS or sLASER). Hence, FB offers a more flexible and reliable choice for high SNR CMRS within the course of 2-3 min. Interestingly, PRESS superior SNR holds potential for reduced acquisition time. The ~ twofold SNR increase when using PRESS allowed to obtain equivalent spectral SNR in a 15 s breath-hold PRESS scan than a 60-heart beats (~ 60 s) free-breathing sLASER (or STEAM) scan. When considering FB spectra drop-outs, the expected fourfold acceleration was not attained. This might be related to the FB retrospective processing, that selected only quality spectra. This processing was not applied to BH acquisitions, in which corrupted spectra might decrease eventual SNR. Retrospective FB spectra selection was chosen as previous findings showed no benefits to employ a prospective respiratory navigator [23]. As expected, both PRESS and semi-LASER acquisitions exhibited phase distortions which commonly reflects VOI displacement (motion) or physiological motion such as blood flow. Semi-LASER data were however strongly affected by peak linewidth and chemical shift distortions, potentially due to exacerbated magnetic susceptibility or gross motion linked to breathing. Proposed retrospective navigation used water signal linewidth and phase to allow reliable and automated gating leading to high SNR spectra. Phase selection was reproduced as previously proposed by Gastl et al. [23]. An additional linewidth threshold was included to further improve the resulting averaged spectrum. The proposed methodology provided reliably high SNR spectra in a finite number of heartbeats. FB also demonstrated excellent clinical sensitivity in another study by Gastl et al. using navigated PRESS sequence with 96 averages to evaluate myocardial triglycerides in healthy volunteers [38]. FB was even pushed further with navigation to acquire metabolite-cycled myocardial MRS in about 13 min [39].

Completing the acquisition protocol, metabolite quantification has been performed, including T1/T2 correction and model fitting. FA concentrations obtained in this study were in line with several reports [7, 26, 31, 37] despite T1/T2 correction not being always explicit for comparisons. Additionally, accurate quantification of myocardial TG and its components requires proper correction for the number of protons per molecule. To the best of our knowledge, only Nakae et al. proposed a correction of the number of protons of the peak signal at 1.2 ppm [7], which is not representative of total myocardial TG [8]. For the purpose of this study and for future quantification we proposed the utilization of the model by Bydder et al. that enables estimation of the number of protons for each specific component of myocardial TG [32]. Conversely, Cr concentrations were lower compared to the literature [30, 31]. This bias might arise from the differences in experimental designs (CMRS sequence, field strength, fitting routine) [1, 29, 31]. Indeed, it stands as one of the first studies measuring myocardial Cr at high field. Our Cr quantification was verified on a phantom containing a calibrated Cr concentration (Additional file 1). Eventually, the sLASER-based Cr quantification proved to be very reliable, thus comforting our results about human myocardial Cr assessment.

## Conclusion

This study proposes a protocol dedicated to in vivo measurements of myocardial metabolism that agrees with the consensus protocols proposed for brain MRS evaluation at 3 T [16]. Cardiac ^1^H-CMRS with sLASER is reproducible and holds potential as a clinical research tool in many cardiac applications. Alternatively, PRESS cardiac ^1^H-CMRS offers increased SNR when acquisition time is scarce, albeit with a slightly reduced reproducibility.

## Supplementary Information

**Additional file 1.** Results obtained on creatine phantom with PRESS, sLASER and STEAMs.

## Data Availability

The datasets used and/or analysed during the current study are available from the corresponding author on reasonable request.

## Abbreviations

BH: Breath-hold
BMI: Body mass index
BW: Bandwidth
CMRS: Cardiovascular magnetic resonance spectroscopy
Cr: Creatine
CSDE: Chemical shift displacement error
ECG: Electrocardiogram
FA: Fatty acids
FB: Free-breathing
FWHM: Full width half maximum
LW: Linewidth
OVS: Outer volume suppression
PRESS: Point resolved spectroscopy
RF: Radiofrequency
SAR: Specific absorption rate
sLASER: Semi-adiabatic Localization by Adiabatic SElective Refocusing
SNR: Signal-to-noise ratio
STEAM: STimulated-Echo Acquisition Mode
SVD: Single value decomposition
TE: Echo time
TG: Triglycerides
TM: Mixing time
TMA: Trimethyl amide
TR: Repetition time
UFA: Unsaturated fatty acids
VOI: Volume of interest
W: Water
