# Supplementary material for: Comparison of single-voxel 1H-cardiovascular magnetic resonance spectroscopy techniques for in vivo measurement of myocardial creatine and triglycerides at 3T
Source: J Cardiovasc Magn Reson. 2021 May 13;23:53. doi: 10.1186/s12968-021-00748-x (PMC8117273; doi:10.1186/s12968-021-00748-x)
Supplement: Supplementary file 1 — Additional file 1. Results obtained on creatine phantom with PRESS, sLASER and STEAMs. [file 12968_2021_748_MOESM1_ESM.docx]

**Additional Material:**

CMRS data were acquired on a homemade phantom containing 10 mmol/L of creatine in agar and water for *in vitro* comparison. The three ^1^H-CMRS sequences PRESS, sLASER and STEAM were used with typical dimensions of 15*20*30mm^3^ with and without water suppression. Quantification of Cr was realized as described in Methods.

|  | **PRESS** | **sLASER** | **STEAM** |
| --- | --- | --- | --- |
| **Cr/W (%)** | 0.034 | 0.030 | 0.023 |
| **[Cr] mmol/L** | 12.70 | 11.26 | 8.54 |

**Suppl. Table 1**. Results obtained on creatine phantom with PRESS, sLASER and STEAMs. Cr content has been measured on a falcon tube containing 10 mmol/L of Cr. Cr/W (%) represents the ratio calculated experimentally on the creatine-containing cylindrical tube; [Cr] represents creatine concentrations measured with each sequence.
